# Development and validation of a clinical prediction model for hospitalization after emergency department admission in patients with cancer

**DOI:** 10.1016/j.esmorw.2025.100141

**Published:** 2025-05-09

**Authors:** Z.L.R. Kaplan, D. van Klaveren, J.G.A. den Duijn, R.J.C.G. Verdonschot, N. Wlazlo, J.G.J.V. Aerts, J. Bromberg, H.F. Lingsma, J. Alsma, M.M.E.M. Bos

**Affiliations:** 1 Department of Public Health, Center for Medical Decision Making, Erasmus University Medical Center, Rotterdam, The Netherlands; 2 Department of Research and Development, Netherlands Comprehensive Cancer Organisation (IKNL), Utrecht, The Netherlands; 3 Department of Medical Oncology, Erasmus University Medical Center, Rotterdam, The Netherlands; 4 Department of Internal Medicine—Section Acute Medicine, Erasmus University Medical Center, Rotterdam, The Netherlands; 5 Department of Emergency Department, Erasmus University Medical Center, Rotterdam, The Netherlands; 6 Department of Hematology, Erasmus University Medical Center, University Medical Center Cancer Institute, Rotterdam, The Netherlands; 7 Department of Pulmonary Medicine, Erasmus University Medical Center, Rotterdam, The Netherlands; 8 Department of Neurology, Erasmus University Medical Center, Rotterdam, The Netherlands

**Keywords:** oncological emergency, clinical prediction model, hospitalization, care efficiency

## Abstract

**Background and purpose:**

Emergency department (ED) admissions by cancer patients often result in hospitalization and prolonged ED stays, contributing to overcrowding. Early identification of patients at risk of hospitalization could improve ED flow and ensure timely provision of oncological care. This study aimed to develop and validate models to predict hospitalization among cancer patients admitted to the ED.

**Methods:**

Adult cancer patients who were admitted to the ED between 1 July 2018 and 30 September 2020 at the Erasmus University Medical Center were included. Data from electronic health records (EHR) were used to develop two logistic regression models: (i) a baseline model including predictors available after ED triage (e.g. patient characteristics, vital parameters) and (ii) an extended model including blood test results. Predictors were selected using the Wald χ^2^ statistic. To prevent overfitting, a uniform shrinkage factor was applied. Model performance was assessed with temporal validation (1 October 2019 to 1 January 2020) and evaluated with calibration plots and C-statistics.

**Results:**

Of 7284 ED admissions, 3967 (54%) resulted in hospitalization. The most common cancers requiring hospitalization were lung, hepatopancreatobiliary, and colorectal cancer. The baseline model included age, sex, primary malignancy, symptoms, metastasis, temperature, pain score, diastolic blood pressure, and heart rate. The model showed good calibration (intercept −0.04, slope 0.86) and discriminative ability [C-statistic 0.71, 95% confidence interval (CI) 0.68-0.74]. The extended model showed improved performance (intercept −0.09, slope 0.92; C-statistic 0.75, 95% CI 0.72-0.78).

**Conclusion:**

Hospitalization risk of cancer patients admitted to the ED can be predicted using routinely collected EHR data, which could aid in optimizing ED patient flow and ensuring timely provision of oncological services.

## Highlights

• Oncological emergencies are characterized by a prolonged ED length of stay and often result in hospitalization.
• Evidence-based risk stratification could support the management of cancer care at the ED.
• Our models can accurately predict hospitalization of patients with cancer after ED admission using EHR data.
• These prediction models could improve ED flow and ensure timely provision of oncological care.

## Introduction

The rapid growth of the cancer population, accompanied by evolving treatment modalities, has significantly increased the burden of cancer-related emergencies on the emergency department (ED).^1^, ^2^, ^3^, ^4^ Acute care is an important aspect of oncological care as it is estimated that almost a quarter of patients with a solid tumor have at least one ED admission during their course of systemic therapy.^5^ Providing efficient oncological care in the ED is, however, challenging due to the wide range of symptoms presented by cancer-related emergencies. These range from the diagnosis and management of treatment-related adverse events (e.g. febrile neutropenia) to patient-centered care issues such as unmanageable symptom control at home.^3^^,^^6^, ^7^, ^8^, ^9^ Consequently, cancer-related ED admissions are more likely to involve acute care conditions and tend to be longer compared with those of the general ED population.^7^^,^^10^, ^11^, ^12^, ^13^

Subsequent hospitalization is often inevitable as more than half of oncological ED admissions result in hospitalization, which is a 3.5 times higher rate compared with non-cancer-related emergency admissions.^6^^,^^7^^,^^10^^,^^14^ This high need of hospitalization combined with prolonged ED stays contributes to ED crowding, which is associated with higher rates of morbidity, increased mortality, and lower patient satisfaction.^15^^,^^16^ Identifying cancer patients at risk of hospitalization early in their ED admission could enhance quality of acute care services by timely transportation to inpatient departments, thereby preventing prolonged ED stays, enabling timely provision of specialized cancer care, and improving patient flow within the ED.^17^, ^18^, ^19^, ^20^ Previous studies have shown that risk of hospitalization increased with age, male patients, patients diagnosed with multiple cancer types, and symptoms related to septicemia.^7^^,^^11^^,^^13^ While several prediction models have been developed to assess hospitalization risk after ED admission, none specifically focus on patients with cancer.^21^

The unique characteristics and health care needs of patients with cancer highlight the urgent need for evidence-based risk assessment tools for cancer-related emergencies.^4^^,^^7^^,^^13^^,^^17^ Therefore, we aimed to develop and validate a model to predict hospitalization of patients with cancer at the ED.

## Methods

### Data

We carried out a retrospective cohort study using electronic health record (EHR) data from the Erasmus University Medical Center (Erasmus MC) in Rotterdam in the Netherlands. The study population consisted of all adult patients with a cancer diagnosis admitted to the ED between 1 July 2018 and 1 January 2020 at the Erasmus MC. For model development we used data from 1 July 2018 to 30 September 2019, and for the temporal validation 1 October 2019 to 1 January 2020. To give an objective view on cancer-related emergency care, our study period was defined as the time after the establishment of the Erasmus MC Cancer Institute and before the start of the coronavirus 2019 pandemic. Patients were retrospectively selected from the EHR based on encoded ‘Diagnosis Treatment Combinations’ (DTC). The DTC is a nationwide coding and reimbursement system providing information on diagnosis and treatment modalities specified by the physician.^22^ For each patient a DTC is opened upon first contact with a physician, who selects a DTC based on specified guidelines. Patients can have multiple DTCs (e.g. multiple primary tumors or other underlying diseases), which only remain active during treatment and follow-up for the specified diagnosis. For the current analysis, all patients with a DTC related to an oncological disease who had an ED admission in the defined study period were included. Potential multiple ED admissions of the same patient were considered as independent admissions. Data collection was carried out by the data capture team of the research suite at Erasmus MC using Castor, the designated platform for research data acquisition.^23^ This study followed the Transparent Reporting of a Multivariable Prediction Model for Individual Prognosis or Diagnosis (TRIPOD) reporting guideline for prognostic studies.^24^ The Daily Board of the Medical Ethics Committee Erasmus MC of Rotterdam, The Netherlands, approved the research proposal (MEC-2022-0674). Of note, patient consent to participate was waived by the Medical Ethics Committee.

### Outcome

The main outcome was hospitalization after ED admission, including those who were transferred to another hospital for further care. To model the need for hospitalization, patients who died at the ED and who went home against medical advice were also considered as hospitalizations. Patients who were transferred to a nursing home were considered discharged. ED discharge status is systematically recorded in the EHR under the aforementioned categories following ED discharge.

### Predictors

Predictor values were extracted from the EHR, consisting of variables systematically recorded by physicians, nurses, and laboratory in predefined fields with specified data entry opportunities. We developed and validated two models for different moments at the ED^1^: a baseline model including all potential predictors available after triage, and^2^ an extended model that additionally included blood test values. Based on prior literature and clinical knowledge we included patient characteristics (i.e. age and sex), disease characteristics (i.e. type of primary malignancy, multiple malignant diagnosis, metastasis, and active systemic treatment) and ED admission characteristics (i.e. mode of transportation and symptoms at presentation) as potential predictors in our model.^6^^,^^7^^,^^11^^,^^13^^,^^21^ The primary malignancy was defined as the first opened DTC in the patient’s history. Multiple malignant diagnosis was defined as the registration of multiple different oncological DTCs following the primary malignancy. Metastasis was defined based on the presence of a DTC specifying metastatic disease status and the type of systemic therapy (i.e. hormonal, immunotherapy, or chemotherapy) administered to patients within 3 months before ED admission, as indicated by treatment codes. The symptoms at ED presentation were routinely registered according to the symptom flowchart of the Manchester Triage System.^25^ Lastly, vital parameters were measured and registered at ED admission [i.e. oxygen saturation, systolic and diastolic blood pressure, heart rate, respiratory rate, body temperature, Glasgow coma scale (GCS) score, and the visual analog scale (VAS) score] and were included as potential predictors. A detailed description of the blood test values is available in the Supplementary Methods, available at https://doi.org/10.1016/j.esmorw.2025.100141. In the case of multiple measurements for the same patient, the first blood test measurement was used. After determining the number of potential predictors, sample size calculations were conducted to analyze the available data support (Supplementary Table S1 available at https://doi.org/10.1016/j.esmorw.2025.100141).

### Model development

Logistic regression analysis was used to analyze the association between the predictors and hospitalization. From the full multivariable model, which included all potential predictors, we selected the final predictors based on Wald χ^2^ statistics (i.e. baseline model *P* < 0.01 and extended model *P* < 0.001), choosing only the most promising predictors for a parsimonious model. We were stricter in variable selection with the extended model to limit the number of predictors in the final model. To enhance the clinical applicability of the model, variable selection was carried out after condensing variables for primary cancer types and symptoms at diagnosis by combining high-risk categories. In the full multivariable model, all continuous predictors were included as restricted cubic splines (three knots). Variable selection of the continuous variables was based on the full variable performing, including all spline parameters. For the final model, the restricted cubic splines were simplified to either linear splines, logarithmic transformations, or quadratic terms. The most suitable transformation for each variable was chosen by comparing the likelihoods and model fit. To prevent overfitting, we estimated a uniform shrinkage factor based on bootstrap validation with a backward selection approach. Shrinkage is necessary to improve model calibration by reducing the variance of the developed model’s predictions in new patients. Subsequently, the regression coefficients of the model were multiplied by the shrinkage factor and the model intercept was adjusted to ensure overall calibration of the model in the validation cohort.^26^ We preferred this modeling strategy over LASSO regression to achieve a balanced combination of model parsimony, interpretability, and predictive ability. All statistical analyses were carried out using R statistical software version 1.4.1103.^27^ Missing predictor values were imputed using a multiple imputation model that included all potential predictors of hospitalization (R-package mice).^28^ The R-package rms was used for the logistic regression analysis.^29^

### Model validation

Model performance was assessed with temporal validation. We assessed the discriminative ability of our models by the area under the curve (AUC; C-statistic) of the receiver operating characteristic (ROC) curve. A C-statistic of 0.5 implies the model is performing no better than random chance, while a C-statistic of 1.0 indicates perfect discrimination. Calibration of the models was assessed with the calibration plot, including the calibration intercept and calibration slope. Ideally, the calibration intercept is zero (i.e. deviation from intercept) and the calibration slope one (i.e. deviation from the average predictor effect).^30^ We additionally estimated the sensitivity and specificity of the models across the entire spectrum of hospitalization risk thresholds.

## Results

### Patient population

The total study population consisted of 7248 ED admissions, which is 15% of all ED admissions between 1 July 2018 and 1 January 2020. Of these, 3967 (54%) admissions required hospitalization, accounting for 24% of all hospitalizations from the ED at the Erasmus MC (Table 1).

**Table 1:** Baseline patient characteristics according to emergency department discharge status for both the derivation and validation cohort

Characteristic	Derivation, *n* = 5997				Validation, *n* = 1287			
	% Missing	Discharged, *n* (%) *n* = 2730 (46%)	Hospitalized, *n* (%) *n* = 3267 (54%)	SMD	% Missing	Discharged, *n* (%) *n* = 587 (46%)	Hospitalized, *n* (%) *n* = 700 (54%)	SMD
**Sex**	0			0.10	0			0.11
Male		1419 (52)	1864 (57)			349 (59)	379 (54)	
Female		1311 (48)	1403 (43)			238 (41)	321 (46)	
**Age, years (IQR)**	0	62 (52-70)	65 (55-71)	0.18	0	63 (52-71)	65 (56-73)	0.18
**Referral place**	8.1			0.10	2.6			0.13
Home		2135 (85)	2563 (85)			506 (88)	594 (88)	
General practitioner		211 (8.4)	198 (6.6)			43 (7.4)	47 (7.0)	
Hospital		114 (4.5)	168 (5.6)			25 (4.3)	23 (3.4)	
Other health care institution		15 (0.6)	33 (1.1)			1 (0.2)	8 (1.2)	
Scene of accident		36 (1.4)	41 (1.4)			3 (0.5)	3 (0.4)	
**Referrer**	14			0.10	3.3			0.02
Self-referral^a^		2122 (89)	2562 (92)			526 (92)	619 (92)	
General practitioner		225 (9.5)	208 (7.4)			44 (7.7)	50 (7.4)	
Other care provider		33 (1.4)	26 (0.9)			2 (0.3)	3 (0.4)	
**Mode of ED arrival**	8.0			0.60	2.5			0.59
Ambulance		432 (17)	1317 (44)			110 (19)	305 (45)	
Own transportation		2071 (83)	1699 (56)			470 (81)	370 (55)	
**ED arrival shift**	0			0.13	0			0.10
Weekday daytime		2165 (79)	2479 (76)			470 (80)	562 (80)	
Weekday nighttime		63 (2.3)	143 (4.4)			16 (2.7)	26 (3.7)	
Weekend daytime		317 (12)	371 (11)			62 (11)	58 (8.3)	
Weekend nighttime		185 (6.8)	274 (8.4)			39 (6.6)	54 (7.7)	
**Emergency severity index**	0.9			0.65	1.1			0.63
Not urgent		985 (36)	458 (14)			182 (32)	93 (13)	
Low urgency		24 (0.9)	4 (0.1)			6 (1.0)	0 (0)	
Urgent		1433 (53)	1926 (59)			309 (54)	360 (52)	
High urgency		258 (9.6)	798 (25)			79 (14)	231 (33)	
Acute life-threatening		0 (0)	58 (1.8)			1 (0.2)	12 (1.7)	
**Lead time, min (IQR)**	0	196 (135-276)	242 (176-330)	0.40	0	207 (139-281)	242 (175-318)	0.33
**Primary cancer type**	0			0.27	0			0.27
Head and neck cancer		207 (7.6)	245 (7.5)			66 (11)	62 (8.9)	
Lung cancer		273 (10)	422 (13)			65 (11)	96 (14)	
Breast cancer		216 (7.9)	138 (4.2)			29 (4.9)	27 (3.9)	
Hepatopancreatobiliary cancer		163 (6.0)	308 (9.4)			38 (6.5)	68 (9.7)	
Colorectal cancer		150 (5.5)	243 (7.4)			32 (5.5)	42 (6.0)	
Nonspecified gastrointestinal cancer		50 (1.8)	108 (3.3)			7 (1.2)	24 (3.4)	
Melanoma		247 (9.0)	237 (7.3)			54 (9.2)	55 (7.9)	
Cancer of bone and connective tissue		68 (2.5)	86 (2.6)			14 (2.4)	14 (2.0)	
Female reproductive cancer		193 (7.1)	203 (6.2)			52 (8.9)	50 (7.1)	
Stomach and esophagus cancer		141 (5.2)	145 (4.4)			25 (4.3)	29 (4.1)	
Central nerve system tumor		169 (6.2)	177 (5.4)			35 (6.0)	42 (6.0)	
Hematological cancer		502 (18)	535 (16)			95 (16)	114 (16)	
Urinary tract cancer		230 (8.4)	298 (9.1)			51 (8.7)	53 (7.6)	
Male reproductive cancer		40 (1.5)	33 (1.0)			9 (1.5)	3 (0.4)	
Prostate cancer		41 (1.5)	42 (1.3)			8 (1.4)	9 (1.3)	
Unspecified malignancy		40 (1.5)	47 (1.4)			7 (1.2)	12 (1.7)	
**Multiple tumors**	0			0.06	0			0.04
One malignancy diagnosed		2378 (87)	2779 (85)			507 (86)	615 (88)	
More than one malignancy diagnosed		352 (13)	488 (15)			80 (14)	85 (12)	
**Metastasis registered**	0			0.09	0			0.10
No metastasis registered		2397 (88)	2773 (85)			520 (89)	596 (85)	
Metastasis registered		333 (12)	494 (15)			67 (11)	104 (15)	
**Systemic treatment**	0			0.08	0			0.11
No systemic treatment		1522 (56)	1919 (59)			317 (54)	398 (57)	
Chemo-immunotherapy		117 (4.3)	121 (3.7)			40 (6.8)	43 (6.1)	
Chemotherapy		574 (21)	666 (20)			124 (21)	141 (20)	
Hormonal therapy		40 (1.5)	27 (0.8)			8 (1.4)	3 (0.4)	
Immunotherapy		477 (17)	534 (16)			98 (17)	115 (15)	
**Prior ED utilization (30 days)**	7.0	433 (17)	667 (22)	0.13	0	105 (18)	148 (21)	0.08
**Prior hospitalization (30) days)**	7.0	180 (7.1)	346 (11)	0.15	0	48 (8.2)	85 (12)	0.13

ED, emergency department; IQR, interquartile range; SMD, standardized mean difference.

a Referral to the hospital is always in consult and not solely based on patient decision.

The development cohort comprised 5997 admissions, with 3276 (54%) resulting in hospitalization. Patients ultimately hospitalized were more often triaged as urgent at presentation [59% versus 53%, standardized mean difference (SMD) 0.60]. We observed high rates of ambulance transportation in the hospitalized patients (44% versus 17%, SMD 0.70) and longer ED length of stay [median 242 min [interquartile range (IQR) 176-330 min] versus 196 min (135-276 min), SMD 0.4]. Patients hospitalized exhibited elevated temperatures (SMD 0.36, Table 2) and elevated infection parameters [i.e. leukocytes (SMD 0.23), C-reactive protein (CRP; SMD 0.53), Table 2]. The most common cancer types resulting in hospitalization were lung cancer (13% versus 10%), hepatopancreatobiliary cancer (9% versus 6%), and colorectal cancer (7.4% versus 5.5%). Similar patterns were observed in our validation cohort, which consisted of 1287 patients, with 700 (54%) requiring hospitalization (Tables 1 and 2).

**Table 2:** Baseline measurements and blood biomarkers according to emergency department discharge status for both the derivation and validation cohort

Characteristic	Derivation, *n* = 5997				Validation, *n* = 1287			
	% Missing	Discharged, *n* = 2730 (46%)^a^	Hospitalized, *n* = 3267 (54%)^a^	SMD	% Missing	Discharged, *n* = 587 (46%)^b^	Hospitalized, *n* = 700 (54%)^b^	SMD
Heart rate (bpm)	43	87 (75-100)	93 (80-109)	0.30	26	88 (77-100)	94 (80-110)	0.30
SBP (mmHg)	12	139 (124-154)	134 (118-150)	0.21	8.9	140 (126-154)	135 (120-152)	0.18
DBP (mmHg)	12	83 (74-93)	80 (70-90)	0.24	9.0	85 (76-94)	80 (70-90)	0.30
Saturation (%)	12	97 (96-98)	96 (95-98)	0.12	8.5	97 (96-98)	96 (95-98)	0.35
RR (/min)	68	16 (14-18)	18.0 (15-22)	0.23	59	16 (14-20)	18 (15-23)	0.32
Temperature (°C)	17	36.9 (36.5-37.2)	37.10 (36.6-37.8)	0.36	12	36.8 (36.4-37.2)	37 (36.6-37.7)	0.36
VAS pain score	5.9	2.00 (0-4)	2 (1-4)	0.10	9.5	2 (0-4)	2 (1-4)	0.17
Glasgow Coma Scale	67	15 (15-15)	15 (15-15)	0.22	75	15 (15-15)	15 (15-15)	0.27
ASAT (U/l)	37	26 (20-39)	29 (21-51)	0.18	34	25 (19-37)	30 (21-50)	0.30
ALAT (U/l)	36	24 (16-40)	26 (17-46)	0.12	34	22 (14-35)	22 (14-45)	0.24
Alkaline phosphatase (U/l)	36	94 (73-134)	111 (80-189)	0.28	35	95 (76-132)	112 (83-176)	0.39
Gamma-GT (U/l)	36	45 (26-92)	68 (34-183)	0.28	34	46 (26-91)	61 (33-154)	0.23
Bilirubin (μmol/l)	36	7 (5-12)	9 (6-15)	0.23	34	7 (5-10)	9 (6-15)	0.23
Albumin (g/l)	94	41 (37-43)	37 (31-42)	0.44	92	32 (28-35)	27 (23-33)	0.55
APTT (s)	92	28 (24-35)	27 (24-33)	0.07	93	28 (24-34)	27 (24-33)	0.24
PT (s)	91	14 (12-25)	14 (12-22)	0.14	92	13 (12-22)	14 (12-25)	0.37
INR	91	1.3 (1.0-2.4)	1.3 (1.1-1.9)	0.06	92	1.2 (1.1-2.1)	1.2 (1.1-2.1)	0.26
Hb (mmol/l)	17	7.5 (6.-8.5)	7.2 (6.1-8.2)	0.26	15	7.6 (6.6-8.4)	7.3 (6.1-8.2)	0.26
Thrombocytes (10^3^/μL)	18	238 (170-313)	243 (166-340)	0.07	15	244 (177-327)	253 (175-351)	0.06
Leukocytes (10^9^/l)	18	7.6 (5.3-10.3)	9.3 (6.2-13.2)	0.23	15	8.0 (5.9-10.3)	9.3 (6.4-13.6)	0.17
MCV (fl)	18	90 (86-95)	90 (86-95)	0.05	14	90 (86-95)	89 (85-94)	0.11
RDW (fl)	43	14.2 (13.1-15.9)	14.7 (13.3-16.5)	0.20	18	14.0 (13.0-15.6)	14.6 (13.3-16.5)	0.28
Hematocrit (l/l)	91	0.4 (0.3-0.4)	0.4 (0.3-0.4)	0.12	91	0.4 (0.3-0.4)	0.4 (0.4-0.4)	0.02
LDH (U/l)	37	232 (193-290)	243 (197-327)	0.11	35	242 (196-300)	256 (201-334)	0.15
ESR (mm/uur)	99	22 (16-46)	28 (16-70)	0.17	99	14 (6-25)	20 (20-69)	0.77
CRP (mg/l)	19	14 (4-45)	41 (10-105)	0.53	16	17 (4-53)	44 (12-108)	0.51
Glucose (mmol/l)	20	6.5 (5.7-7.7)	7.1 (6.1-8.9)	0.27	18	6.4 (5.7-7.7)	6.9 (5.9-8.4)	0.21
Calcium (mmol/l)	36	2.4 (2.3-2.5)	2.3 (2.2-2.4)	0.15	35	2.4 (2.3-2.5)	2.3 (2.2-2.4)	0.04
Sodium (mmol/l)	18	139 (136-141)	137 (134-140)	0.38	16	139 (137-141)	137 (134-140)	0.38
Potassium (mmol/ml)	19	4.1 (3.9-4.4)	4.1 (3.8-4.5)	0.03	17	4.2 (3.9-4.4)	4.1 (3.8-4.5)	0.0
Troponin (μg/l)	92	14 (9-22)	30 (15-61)	0.29	91	11 (6-21)	40 (18-65)	0.73
NT-proBNP (pg/ml)	98	118 (38-342)	321 (68-901)	0.57	96	81 (11-221)	135 (64-486)	0.47
eGFR (ml/min)	18	82 (63-97)	77 (54-95)	0.21	15	82 (60-96)	77 (51-95)	0.18
Urea (mmol/l)	35	5.6 (4.2-7.4)	6.6 (4.8-9.5)	0.39	34	5.9 (4.4-8.0)	6.9 (4.9-10.8)	0.34
Creatinine (μmol/l)	18	79 (65-98)	84 (66-112)	0.13	15	80 (67-103)	83 (65-112)	0.14

ALAT, alanine aminotransferase; APTT, activated partial thromboplastin time; ASAT, aspartate aminotransferase; CRP, C-reactive protein; DBP, diastolic blood pressure; eGFR, estimated glomerular filtration rate; ESR, erythrocyte sedimentation rate; INR, international normalized ratio; LDH, lactate dehydrogenase; MCV, mean corpuscular volume; NT-proBNP, N-terminal pro B-type natriuretic peptide; PT, prothrombin time; RDW, red cell distribution width; RR, respiratory rate; SBP, systolic blood pressure; SMD, standardized mean difference; VAS, visual analog scale.

**Figure 1:**
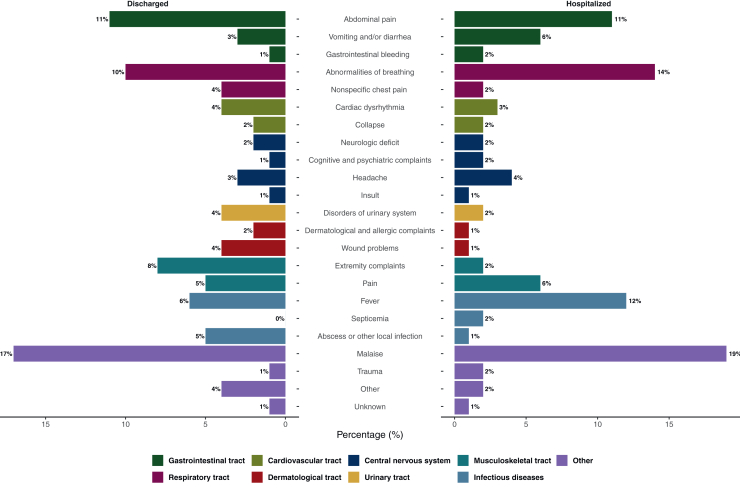
**Symptoms registered at presentation.** This figure illustrates the symptoms registered at emergency department presentation for both the discharged and hospitalized patient populations, encompassing both the derivation and validation cohorts. Symptom documentation is systematically carried out according to the Manchester Triage System. Symptoms are further categorized based on their anatomical tract.

In both groups, abdominal pain (11%), abnormalities in breathing (10% versus 14%), and malaise (17% versus 19%) were the most common symptoms at presentation (Figure 1; Supplementary Table S2, available at https://doi.org/10.1016/j.esmorw.2025.100141). Hospitalized patients presented more frequently with vomiting and/or diarrhea (6%), gastrointestinal bleeding (2%), pain (6%), cognitive and psychiatric complaints (2%), and septicemia (2%) (Figure 1). See further details in Supplementary Table S3 available at https://doi.org/10.1016/j.esmorw.2025.100141).

### Prediction of hospitalization

**Figure 2:**
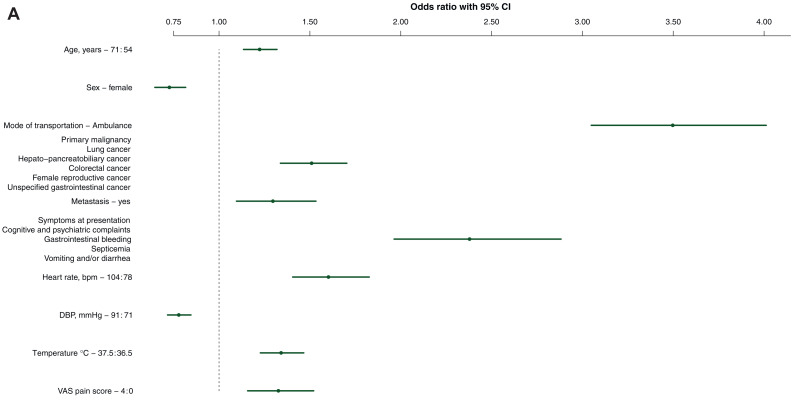

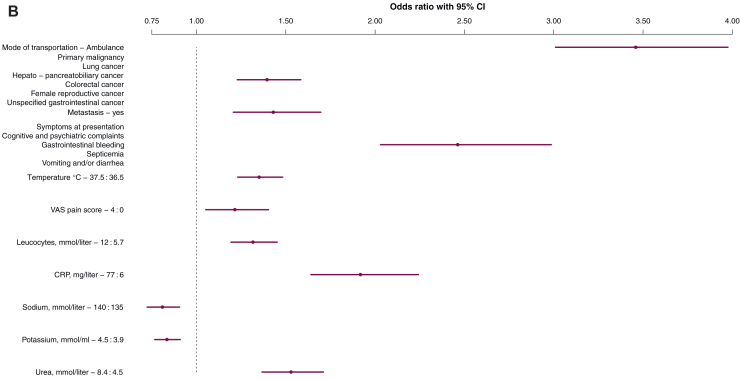
**Forest plots illustrating the odds ratios with 95% confidence intervals (CIs) for the final predictors of hospitalization in the baseline model (A) and the extended model (B).** Coefficients for the continuous variables are calculated using the interquartile ranges of the total population. CRP, C-reactive protein; DBP, diastolic blood pressure; VAS, visual analog scale.

**Figure 3:**
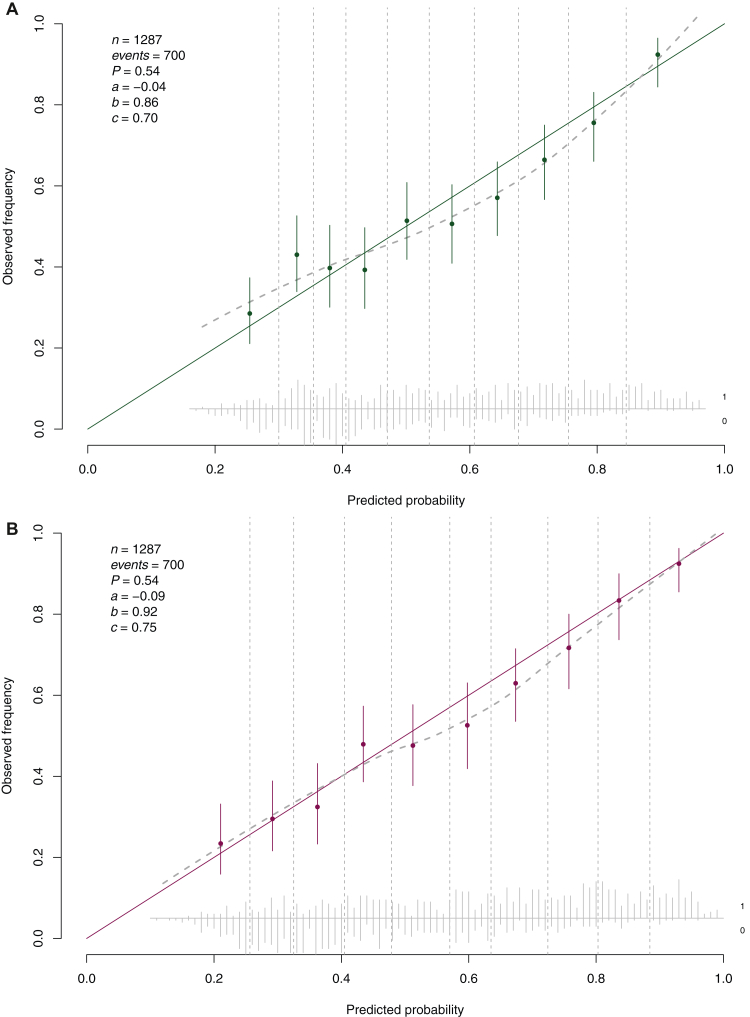
**This figure illustrates the calibration plots for both the baseline model (A) and the extended model (B) used to predict hospitalization within our validation cohort.** The validation cohort comprises patients admitted to the emergency department between 1 October 2019 and 1 January 2020. Parameters include *n* for the number of patients; *a* for the calibration intercept (0 indicates perfect calibration); *b* for the calibration slope (1 indicates perfect calibration); *c* for the area under the curve (AUC; 0.5 indicates chance performance, while 1 indicates perfect performance).

The predictive ability of the baseline model was primarily driven by mode of transportation (χ^2^ = 320), temperature (χ^2^ = 128), and symptoms at presentation (χ^2^ = 78). The model also included heart rate (χ^2^ = 52), primary malignancy (χ^2^ = 45), diastolic blood pressure (χ^2^ = 43), VAS pain score (χ^2^ = 40), sex (χ^2^ = 29), age (χ^2^ = 28), and presence of metastasis (χ^2^ = 9) as final predictors of hospitalization (Table 3; Figure 2A, Supplementary Table S4, available at https://doi.org/10.1016/j.esmorw.2025.100141). All vital parameters were transformed using linear splines (Supplementary Figure S1, available at https://doi.org/10.1016/j.esmorw.2025.100141), and a uniform shrinkage factor of 0.94 was estimated. Our final baseline model demonstrated good discrimination within our validation cohort, achieving an AUC of 0.70 [95% confidence interval (CI) 0.67-0.73]. Additionally, the model exhibited acceptable calibration, as indicated by a calibration intercept of −0.04 (95% CI −0.2 to 0.08) and a calibration slope of 0.87 (95% CI 0.73-1.01) (Figure 3A).

**Table 3:** Presentation of final models

Predictor^a^	OR	95% CI	Wald χ^2^	OR after shrinkage^b^
Baseline model				
**Age, years (71 versus 54)**	1.22	1.13-1.32	28	1.21
**Sex**				
Male		(ref)		
Female	0.73	0.65-0.82	29	0.74
**Mode of transportation**				
Own transportation		(ref)		
Ambulance transportation	3.50	3.05-4.01	320	3.26
**Primary malignancy**				
All other malignancies		(ref)		
Lung cancer	1.51	1.34-1.70	45	1.48
Hepatopancreatobiliary cancer				
Colorectal cancer				
Female reproductive cancer				
Unspecified gastrointestinal cancer				
**Metastasis registered**				
No		(ref)		
Yes	1.30	1.10-1.53	9	1.28
**Symptoms at presentation**				
**All other symptoms**		(ref)		
Cognitive and psychiatric complaints	2.38	1.96-2.88	78	2.27
Gastrointestinal bleeding				
Septicemia				
Vomiting and/or diarrhea				
**Heart rate (bpm) (104 versus 77)**	1.60	1.41-1.83	52	1.56
**DBP (mmHg) (91 versus 71)**	0.78	0.72-0.85	43	0.79
**Temperature (°C) (37.5 versus 36.5)**	1.34	1.23-1.47	128	1.32
**VAS pain score (4 versus 0)**	1.33	1.16-1.52	40	1.31

**Extended model**				

**Mode of transportation**				
Own transportation		(ref)		
Ambulance transportation	3.46	3.01-3.98	306	3.19
**Primary malignancy**				
All other malignancies		(ref)		
Lung cancer	1.40	1.23-1.58	26	1.36
Hepatopancreatobiliary cancer				
Colorectal cancer				
Female reproductive cancer				
Unspecified gastrointestinal tumors				
**Metastasis registered**				
No		(ref)		
Yes	1.43	1.21-1.70	17	1.40
**Symptoms at presentation**				
All other symptoms		(ref)		
Cognitive and psychiatric complaints	2.46	2.03-2.99	84	2.32
Gastrointestinal bleeding				
Septicemia				
Vomiting and/or diarrhea				
**Temperature (°C) (37.5 versus 36.5)**	1.35	1.23-1.48	126	1.32
**VAS pain score (4 versus 0)**	1.21	1.05-1.40	22	1.20
**Leukocytes (mmol/l) (12 versus 5.7)**	1.32	1.19-1.45	32	1.29
**CRP (mg/l) (77 versus 6)**	1.92	1.64-2.24	125	1.84
**Sodium (mmol/l) (140 versus 135)**	0.81	0.72-0.91	40	0.82
**Potassium (mmol/ml) (4.5 versus 3.9)**	0.83	0.77-0.91	22	0.84
**Urea (mmol/l) (8.4 versus 4.5)**	1.53	1.37-1.71	55	1.49

CI, confidence interval; CRP, C-reactive protein; DBP, diastolic blood pressure; OR, odds ratio; ref, reference; VAS, visual analog scale.

a Coefficients for the continuous variables are calculated using the interquartile ranges of the total population.

b This row represent the adjusted odds ratio with shrinkage, baseline model 0.94 and the extended model 0.93.

The predictive ability of our extended model was primarily driven by mode of transportation (χ^2^ = 306), temperature (χ^2^ = 126), CRP (χ^2^ = 125), and symptoms at presentation (χ^2^ = 84). The extended model also included urea (χ^2^ = 55), sodium (χ^2^ = 40), leucocytes (χ^2^ = 32), primary malignancy (χ^2^ = 26), VAS pain score (χ^2^ = 22), potassium (χ^2^ = 22), and presence of metastasis (χ^2^ = 17) (Table 3; Figure 2B, Supplementary Table S5, available at https://doi.org/10.1016/j.esmorw.2025.100141). In the extended model, urea was transformed using a natural logarithm and the remaining continuous variables with a linear spline (Supplementary Figure S2, available at https://doi.org/10.1016/j.esmorw.2025.100141). We additionally applied a shrinkage factor of 0.93. Compared with our baseline model, the extended model exhibited improved discriminative ability with an AUC of 0.75 (95% CI 0.73-0.78) and better calibration, reflected by a calibration intercept of −0.09 (95% CI −0.21 to 0.04) and a calibration slope of 0.92 (95% CI 0.79-1.06) (Figure 3B). In Supplementary Figure S3, available at https://doi.org/10.1016/j.esmorw.2025.100141, the sensitivity and specificity across the entire spectrum for hospitalization risk threshold is shown. Clinical decision making based on our model requires deciding on a cut-off to admit patients. That cut-off can be determined per setting and is dependent on the desired sensitivity and specificity. For example, if we accept a threshold of 75%, the sensitivity is 39% and the specificity 91%.

### Model presentation

The resulting models were implemented as a publicly accessible web-based application. According to the TRIPOD checklist (Supplementary Material, available at https://doi.org/10.1016/j.esmorw.2025.100141), all relevant items are covered in this manuscript, except for the availability of data sets.

## Discussion

Hospitalization of patients with cancer after an ED admission can be predicted using routinely collected data from the EHR. We have developed and validated two prediction models, both demonstrating good discriminative ability and acceptable calibration. These models could function effectively in risk-stratifying patients for hospitalization and optimizing patient flow within the ED, ultimately ensuring the timely provision of acute oncological services.

In our study, patients with cancer comprised nearly 15% of all ED admissions, a significantly higher proportion compared with population-based data where cancer patients typically make up 3%-4% of ED admissions.^7^^,^^10^^,^^31^^,^^32^ The primary reason is likely attributed to the academic nature of the Erasmus MC, which serves as a specialized tertiary referral center within its region, particularly for managing complex cases, including acute oncology. For example, during our study period, the administration of immunotherapy was limited to academic and large peripheral hospitals, whereby complications arising from therapy were managed by these centers. Additionally, it should be acknowledged that this elevated percentage could also be a reflection of a greater demand for cancer-related emergency care and longer survival durations among cancer patients.^1^^,^^2^

Previous studies have highlighted lung, colorectal, prostate, and breast cancer as the most common types leading to ED admission, which is most presumably a reflection of the population prevalence rates of these common malignancies.^2^^,^^6^^,^^7^^,^^10^^,^^32^ However, in our study, we found that hematological cancer, lung cancer, urinary tract cancer, and melanoma were the most frequent causes of ED admission. In comparison with other malignancies, a large proportion of these patients received intensive systemic therapy, including immunotherapy (e.g. adoptive cell therapy and immune checkpoint inhibitors as CTL-4 and PD-L1 antibodies), which could lead to malaise and gastrointestinal issues.^33^, ^34^, ^35^ Of note, the high proportion of ED admissions by hematological cancer patients can additionally be attributed to reduced immunity from stem cell transplantation and treatment with CAR T cells, as well as to the relatively large hematology department of our center.^36^

Our study showed that more than half of the ED admissions resulted in hospitalization, which closely aligns with prior research. According to our models, the risk of hospitalization was the highest among patients with lung cancer, hepatopancreatobiliary cancer, colorectal cancer, cancer of the female reproductive system, and nonspecified abdominal cancers. This is expected, as these cancer types necessitate complex, high-risk treatments (i.e. targeted therapy and abdominal surgery), which are prone to complications and could necessitate urgent care.^34^^,^^36^, ^37^, ^38^ Additionally, the majority of patients with these malignancies are often diagnosed in late stages, more often with metastases resulting in the need for critical care, and also in the palliative phase, due to complaints of breakthrough pain, cognitive symptoms, and malaise.^2^^,^^39^ Our model additionally revealed that infection issues, cognitive symptoms, and gastrointestinal problems constituted high risk for hospitalization, which are likely a reflection of the immunosuppressive effects of cancer and its treatments and tumor-related organ involvement.^11^

In recent years, various prognostic tools have been developed for the general ED population, aimed at predicting hospitalization and enhancing patient flow.^21^^,^^40^ While these models could potentially be applied to cancer patients, there is a notable disparity in outcome proportions, with hospitalization rates often exceeding 50%.^6^^,^^7^^,^^10^ Our baseline model is readily usable upon patient arrival following triage. Utilizing this model, patients at high risk of hospitalization can be promptly admitted without the need for further assessment in the ED. Additionally, these probabilities can be helpful in managing patient expectations upon arrival, potentially improving their experience in the ED.^41^ The second model, incorporating blood test values, enhances discrimination and exhibits better calibration. The blood test values were found to be an important prognostic factor, alleviating the association between demographic characteristics (i.e. sex and age) and outcome that we observed in our baseline model. While predictions are more accurate with this model, an initial ED work-up is still necessary for its utilization.

While our prediction models can significantly enhance the efficiency of acute oncological care, prevention of ED admissions and subsequent hospitalizations is equally crucial. For instance, a recent report estimated that, based on discharge diagnoses, nearly half of ED admissions could potentially be prevented. Of note, ∼30% of these avoidable admissions led to hospitalization.^42^ Nevertheless, identifying preventable ED admissions based solely on discharge diagnosis is complex and somewhat arbitrary, as ED utilization by patients with cancer is multifactorial.^43^ ED utilization is influenced by interactions between individual capabilities (e.g. self-care proficiency and access to health care services), provider attributes (e.g. knowledge, communication skills, and referral practices), and health system dynamics (e.g. bed availability, staffing levels, and access to social support services).^44^ Thereby many potentially avoidable ED admissions leading to hospitalization can be attributed to significant patient delays, often caused by a lack of self-care proficiency or insufficient information and communication from caregivers in the outpatient setting. Ideally, such admissions could be avoided through better patient education, modern communication strategies, and early intervention at outpatient clinics, addressing patient concerns before they escalate to the point of requiring ED care and eventual hospitalization.^42^^,^^44^^,^^45^ This complex interplay, therefore, requires a multidisciplinary approach with multiple interventions also targeting prevention.^45^

### Limitations

The findings of this study must be interpreted considering several limitations. Firstly, our model was developed using data from a single center, potentially limiting its generalizability despite temporal validation. Secondly, given our reliance on registered EHR data, there exists a potential for registration bias, particularly concerning the documentation of symptoms at ED presentation, multiple tumors, and metastases. For example, malaise was a common symptom among both discharged and hospitalized patients. Given the lack of further distinction regarding this symptom, its specification and registration according to the Manchester Triage System is essential for better understanding and management of this patient population. Lastly, health care utilization is dependent on social factors, as described by the Andersen model, which includes predisposing factors (e.g. health beliefs), enabling factors (e.g. personal resources, community resources), and need factors (e.g. perceived and evaluated need for health services).^46^ These factors, which potentially influence care decisions, were not included in our models.

### Conclusion

The acute care management of cancer patients demands evidence-based interventions tailored specifically for the ED setting. In this context, our study offers a data-driven approach through the introduction of two prediction models demonstrating good discrimination and calibration. These models effectively identify patients at risk of hospitalization, facilitating early intervention and enhancing ED workflow and efficiency. Furthermore, they can serve as valuable, informative tools for managing patient expectations during their ED experience. Given that both models rely on routinely collected predictors within hospital settings, they are ready to integrate into the electronic health system. Moving forward, efforts should be directed toward the external validation of these models, followed by their implementation across health care facilities. Subsequent evaluation of their clinical utility will be essential in assessing their impact on patient care and outcomes, ultimately advancing the standard of cancer management in acute care settings.
