# Supplementary figures and images for: Development and validation of a clinical prediction model for hospitalization after emergency department admission in patients with cancer
Source: ESMO Real World Data Digit Oncol. 2025 May 9;8:100141. doi: 10.1016/j.esmorw.2025.100141 (PMC12836634; doi:10.1016/j.esmorw.2025.100141)

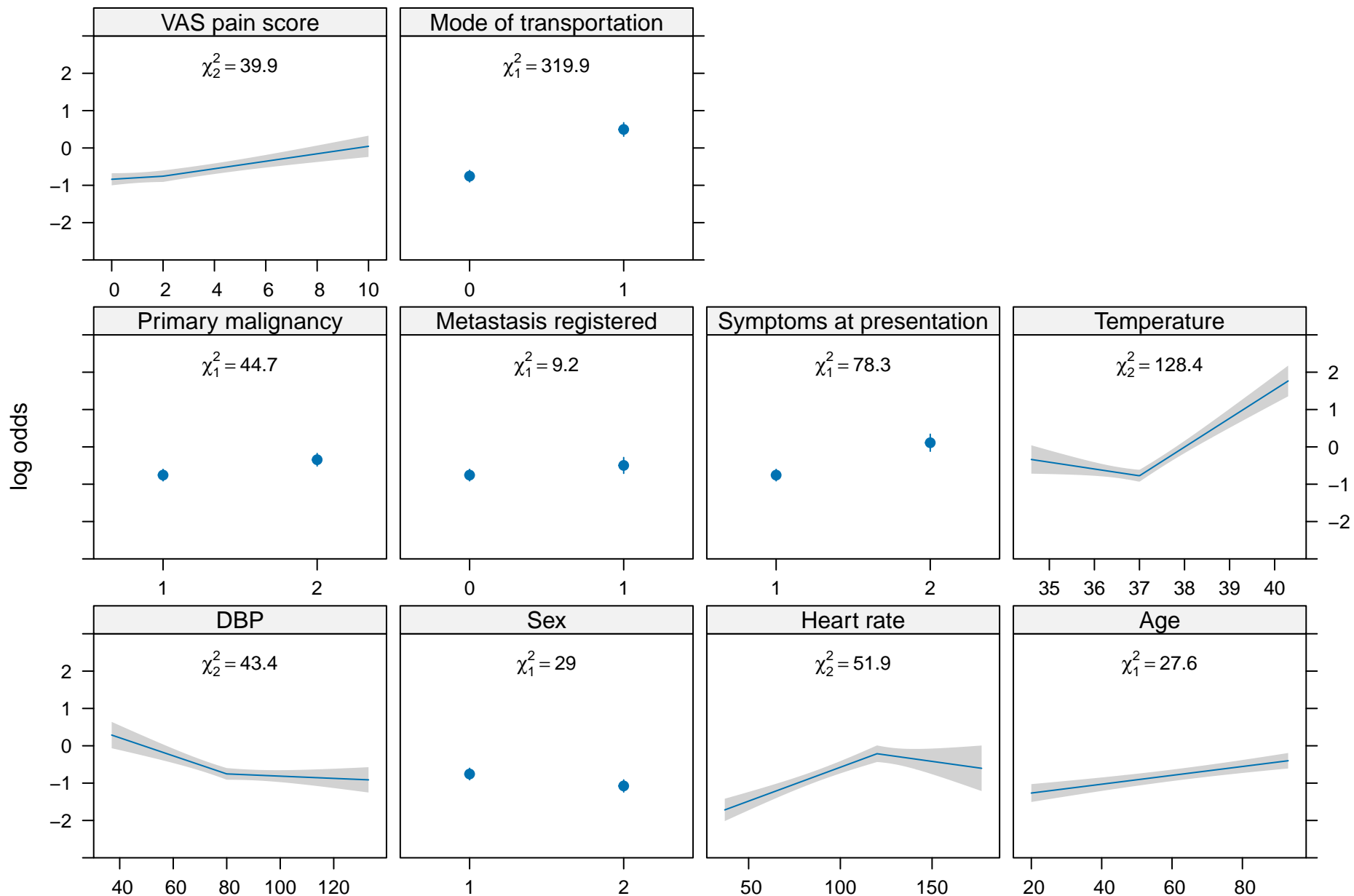

Supplement: Supplementary Figure 1 [file mmc1.pdf]

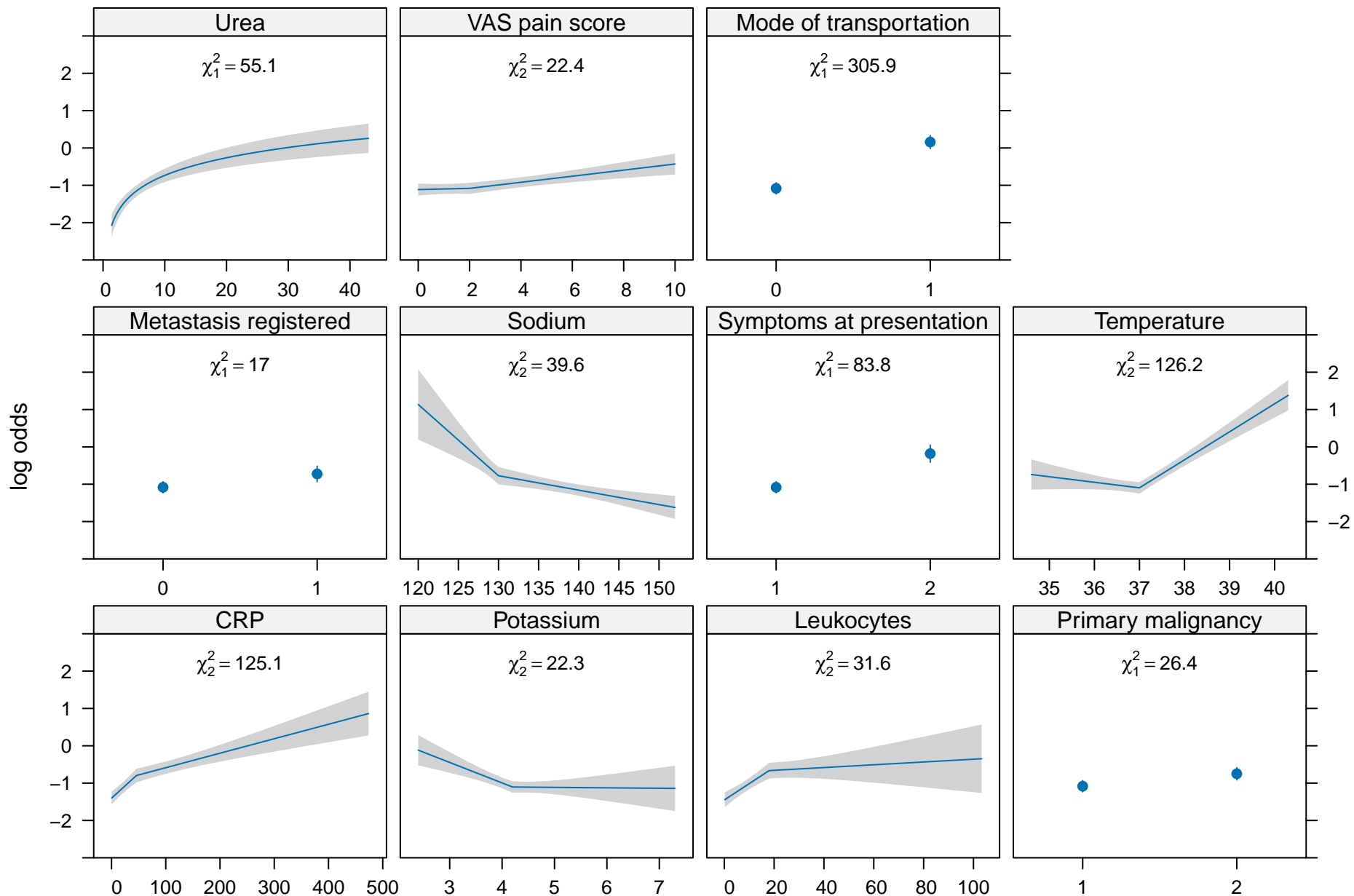

Supplement: Supplementary Figure 2 [file mmc2.pdf]

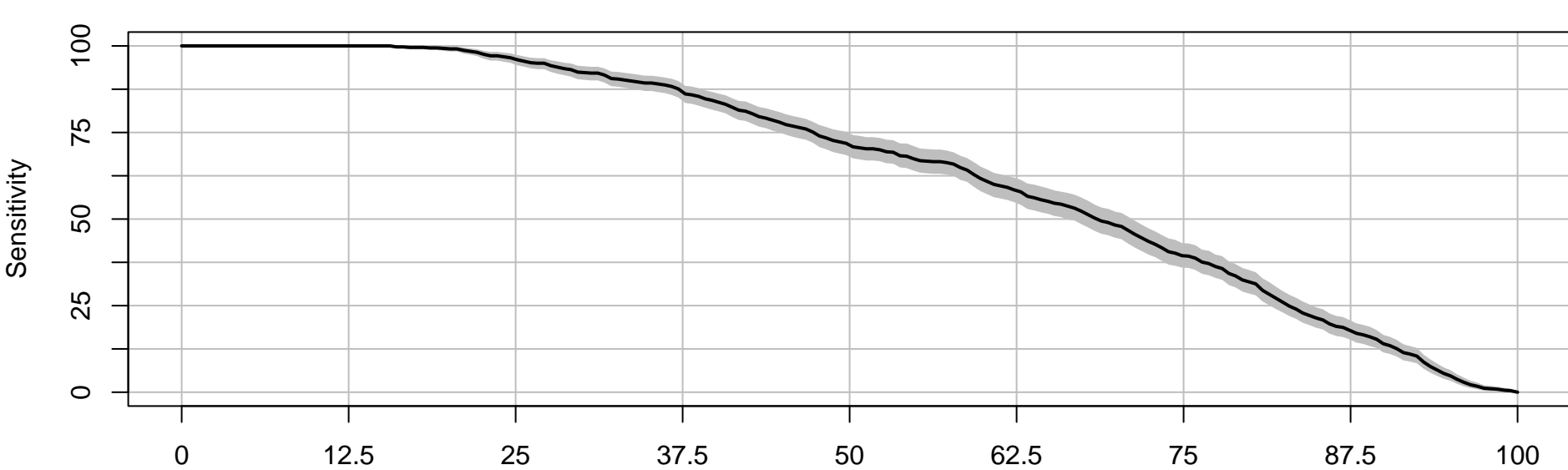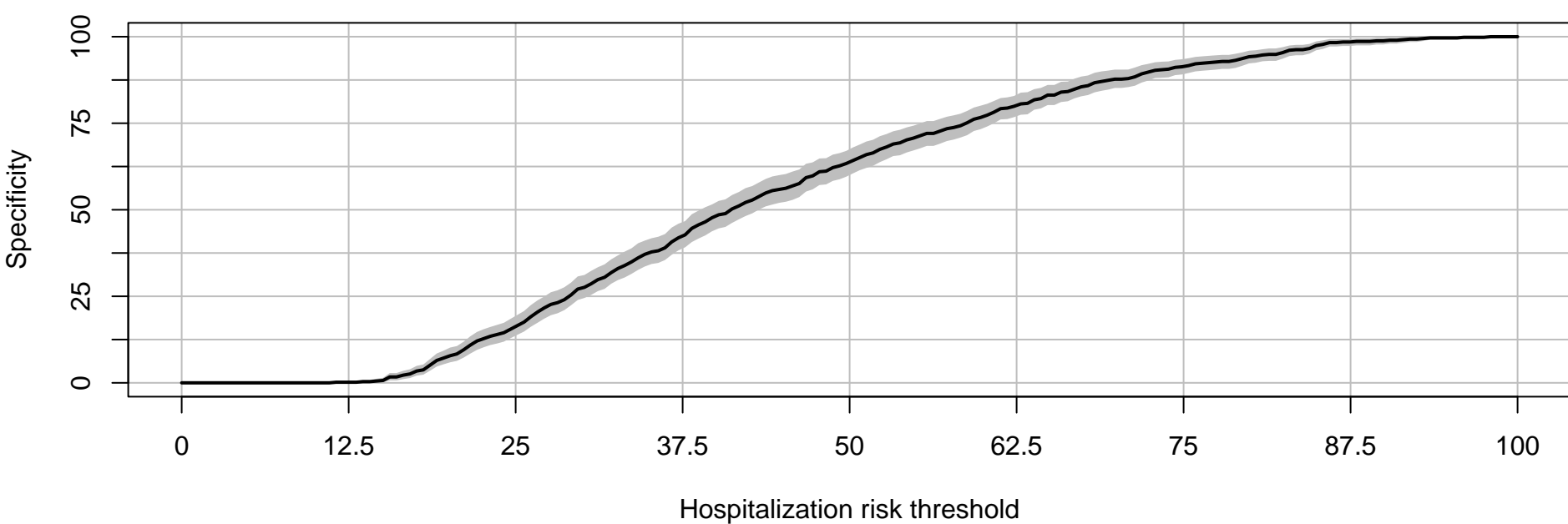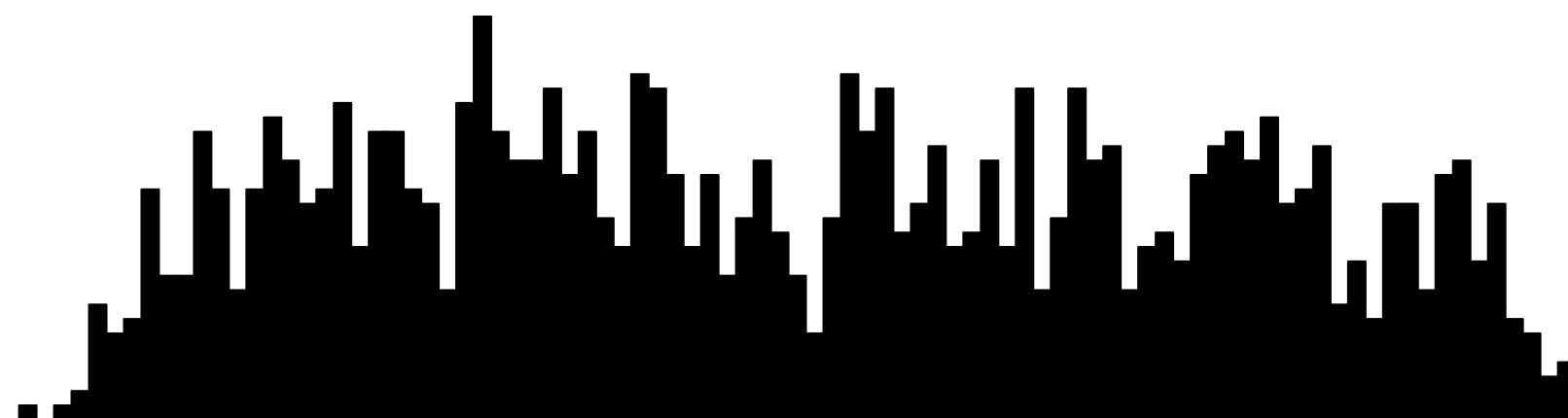

Supplement: Supplementary Figure 3 [file mmc3.pdf]
